# Survival from multiple myeloma in England and Wales up to 2001

**DOI:** 10.1038/sj.bjc.6604608

**Published:** 2008-09-23

**Authors:** S Schey

**Affiliations:** 1 Department of Haematology, King's College Hospital, Denmark Hill, London SE5 9RS, UK

The study of myeloma survival reported by Gahrton *et al* (2001) in this issue of the *British Journal of Cancer* is both enlightening and challenging to clinicians managing this condition. Awareness of myeloma has been increased over the last few years as more refined diagnostic criteria (Rajkumar *et al*, 2007), improved investigational techniques (Ng *et al*, 2006) and better treatments have become available. Myeloma incidence increases with age, (Phekoo *et al*, 2004) but even allowing for an increasingly ageing UK population there seems to be an increased incidence in younger patients, the cause of which is unknown.

Myeloma is a heterogeneous disease clinically, phenotypically and genetically. Some patients present with an indolent smouldering disease whereas in others it is rapidly progressive presenting with painful bony lesions, fractures and hypercalcaemia, neurological damage, renal impairment and myelosuppression. Improvements in supportive therapy, such as plasmapheresis, bisphosphonates (Ashcroft *et al*, 2003) and vertebroplasty (Kose *et al*, 2006), and antimicrobials for fungal and viral infections have improved the quality of life for patients with myeloma.

Despite, advances in our understanding of the biology of multiple myeloma, the disease remains incurable with conventional treatment. Gahrton *et al* (2001) have shown that relative survival, that is, the comparison of survival of individuals with the disease relative to that of all individuals of the same age and sex in the population from which they were drawn, has improved over the period 1986–1999. The introduction of melphalan in the 1960s (Speed *et al*, 1964) allowed for better disease control. With chemotherapy, however, less than 5% of patients achieve a complete remission (CR), median overall survival (OS) is 3–4 years and only 10–15% of patients survive more than 10 years, with the disease inevitably relapsing and patients dying secondary to disease progression and complications.

Although dose escalation and combination chemotherapy improved response rates and disease-free interval in patients younger than 65 years, it has failed to significantly impact on survival in patients aged more than 65 years (Maclennan *et al*, 1992). Two recent population studies from Sweden (Kristinsson *et al*, 2007) and SEERS in the United States (Brenner *et al*, 2008) have shown significantly improved 5- and 10-year survival, but in both studies this improvement has been confined to those under 60 years of age. Data related to patients diagnosed up to 2003 and 2004, respectively, suggesting that the improved survival in those aged less than 60 years is because of the impact of autologous stem cell transplantation, which is confined in the main to those aged under 65–70 years. The impact of new agents at presentation and relapse on long-term survival are yet to be seen, these agents having only come into use after 2000. As the efficacy seems to be similar in all age groups, similar improved survival is to be expected in those more than 60 years of age in the years to come.

Haematopoietic stem cell transplantation increases complete response rates to 40% (Badros *et al*, 2002; Child *et al*, 2003) and significantly prolongs progression-free survival but relapses continue to occur with no plateau in the survival curves (Badros *et al*, 2002). Overall, however, survival rates have been inconsistent among studies. The IFM study (Attal *et al*, 1996) was the first to demonstrate superiority of autologous stem cell transplantation over conventional chemotherapy and the MRC Myeloma VII trial (Child *et al*, 2003) later confirmed this, reporting OS rates of 52–54 months. Another French study (Fermand *et al*, 1998) confirmed the benefit for event-free survival (EFS) but not OS, whereas two other studies (Blade *et al*, 2005; Barlogie *et al*, 2006) both showed significantly increased CR rates but no prolongation in EFS or OS. Differences in the design of the control treatment arms of these studies may account for some of the discrepancies reported and the benefits of autologous transplantation remain controversial (Blade *et al*, 1996). Furthermore, the median age for transplantation in all major clinical trials is between 49 and 52 years, implying that approximately 50% of patients are ineligible for this treatment strategy. Similarly, tandem autologous transplantation increases complete remission rates but has had a disappointing impact on survival. Randomised trials of allogeneic stem cell transplantation are lacking but registry data and small single centre studies suggest this approach may induce prolonged disease-free survival in selected individuals but at the cost of high treatment-related mortality. This has fallen from 40 to 50% for patients reported to the European Blood and Bone Marrow Transplant Register between 1989 and 1991 to 30% for those transplanted between 1995 and 1999 (Gahrton *et al*, 1997). Registry data (Bjorkstrand *et al*, 1996) shows a complete response rate at 5 years of 9% in allogeneic transplantation and 4% in autografting. Maximum duration of complete remission was 113 months for allogeneic *vs* 38 months for autologous transplantation with continuing late relapses after CR up to 91 months in allogeneic patients and 38 months in autologous transplant patients. Non-myeloablative, reduced intensity conditioning (RIC) stem cell transplantation offers the opportunity of reduced mortality although retaining the potential graft *vs* myeloma immunological effect. It has extended the upper age for application of transplantation from 55 years for standard allogeneic transplantation to 70 years for RIC transplantation (Peggs *et al*, 2002). The role of autologous stem cell transplantation followed by a RIC allogeneic stem cell transplant in newly diagnosed myeloma has recently been compared with tandem autologous stem cell transplantation with a significant improvement in EFS and OS for the RIC allograft arm (35 *vs* 29 months, *P*=0.02 and 80 *vs* 54 months, *P*=0.01, respectively) (Bruno *et al*, 2007). Once a patient relapses, there is no consensus on the most suitable therapeutic regimen for re-treatment and the next UKMF/NCRI MM X (Intensive) trial is designed to investigate re-induction using a bortezomib chemotherapy combination and to compare the benefit of consolidation with a second stem cell transplant *vs* further conventional chemotherapy.

As we have entered the new millennium, the poor results of chemotherapy underscore the need for effective therapeutic strategies to improve survival in multiple myeloma. Although the aetiology of the condition remains unknown, the pathogenesis has been intensively investigated, providing insight into the genetic and molecular evolution of the disease and identifying a number of new therapeutic targets (Mitsiades *et al*, 2007). The biological, genetic and molecular variants of myeloma cells lead not only to clinical diversity but also to differences in response to treatment. The importance of cytokines (eg, interleukin IL-6, IL-1*β*, IL-10, and tumour necrosis factor-*α*) and the role of cross talk between myeloma cells and the bone marrow microenvironment in the proliferation, apoptosis and migration of myeloma cells and patient survival is becoming clearer (Chauhan *et al*, 1996; Gupta *et al*, 2001). Small molecules designed to interrupt cellular signalling pathways have led to the development of a variety of new therapies (Corral *et al*, 1999). If these agents are to impact significantly on myeloma survival, there is a need to investigate their use, particularly in patients aged more than 65 years with poor clinical and biological prognostic features (eg, del 13, *t*(4:14) translocations and del 17p53) and in patients with renal failure (Chanan-Khan *et al*, 2007). The outlook for relapsed disease has been significantly improved in early trials using the new agents bortezomib (Rajkumar *et al*, 2005) and the immunomodulatory drugs (Richardson *et al*, 2002; Schey *et al*, 2004; Dimopoulos *et al*, 2005) both as a single agent and in combination with steroids and chemotherapy agents. Trials will need to be designed to address the optimal timing, scheduling and combinations of these agents with conventional chemotherapy, to ensure that overall survival is improved, in addition to improving quality of life by enhancing progression-free survival. Another exciting area, yet to be fully exploited, is the effect of the immune system in the minimal residual disease setting and the role of the immunomodulatory drugs may prove to be of particular interest in this situation. (Davies *et al*, 2001; Dredge *et al*, 2002)

The widening differences in survival between socioeconomic groups remain a concern. The improvements in survival in patients aged 65 years and under, reported in 1990 and 2000 from SEER and Europe, suggests that autologous stem cell transplantation has impacted significantly on survival over this period. The high costs of haematopoietic stem cell transplantation from blood or bone marrow presents a challenge for health care providers. Data on 113 827 patients from 580 centres in 35 European countries between 1990 and 1999 (Gratwohl *et al*, 2002) suggested that the increase in the number of patients receiving transplants annually across Europe between 1990 and 1999 correlated with economic factors. A further study from North America (Mitchell *et al*, 1997) concluded that there was substantial variation in access to bone marrow transplantation for black patients and that they and those enroled in health maintenance organisations, those covered by Medicaid, and self-pay patients were less likely to receive a transplant. Myeloma is almost twice as common in the African-Caribbean population and incidence increases significantly with age. These two groups account for a large proportion of socioeconomically deprived individuals in UK society. Patient performance status and comorbidity plays a major role in patient selection for transplantation and as comorbid problems, such as chronic obstructive pulmonary disease and heart disease are more frequently associated with low socioeconomic status and increasing age; this may adversely impact on patient access to this form of treatment, particularly in the 60–70 years age group.

There may, in addition, be other reasons why these groups have restricted access to effective therapy. Some people may shun conventional medical treatment for more traditional remedies, only seeking medical help late in the course of their disease. Work is needed to gain a better understanding of the cultural attitudes of the African-Caribbean population towards cancer and its treatment, and to target education and information using appropriate channels. Elderly patients are less able or willing to travel to receive treatment, either in specialised units or to access clinical trials. Socioeconomically deprived groups may lack access to information on the world-wide web, which could also be a factor in limiting access to new, more effective treatments.

We are embarking on a new era in the treatment of myeloma where the challenge for the next 10 years is to learn how to maximise the therapeutic advantage of new agents that are rapidly coming on line and translate the hope of the last 5 years into improved outcomes for patients. This can only be done by conducting well-designed clinical trials that are capable of informing not just patients and clinicians of clinical outcomes, but also healthcare planners, enabling them to allocate funding so that the best treatments can be accessed by every patient.
